# Assessment of the alcohol consumption among outdoor bar drinkers in Nigeria by qualitative methods

**DOI:** 10.1186/s12889-018-5250-y

**Published:** 2018-03-06

**Authors:** Victor O. Lasebikan, Olatunde Ayinde, Mayokun Odunleye

**Affiliations:** 1 Department of Psychiatry, College of Medicine, University of Ibadan, PMB, Ibadan, 5116 Nigeria; 2 Department of Psychiatry, University College Hospital, Ibadan, Nigeria

**Keywords:** Outdoor bars, Problem drinkers, Focus group discussion, Direct observation, Open display of alcohol

## Abstract

**Background:**

There are indications that drinking in outdoor bars, such as at motor-parks, by the roadsides or street corners have become popular in Nigeria.

**Method:**

Three sets of qualitative assessments were carried out from three outdoor bars, randomly selected from 22 of such in Ibadan, Nigeria. The main sources of data were by direct observation and focus group discussion (FGD), conducted by a non-probabilistic sample of outdoor bar drinkers, alcohol vendors and from community members. The qualitative assessments were recorded, followed by a thematic analysis of the contents of the qualitative assessments.

**Results:**

Widespread use of alcohol was reported. Patrons of outdoor bars reported that their context of drinking was pleasurable to them. Use of local beverages usually called ‘sepe’ is increasing. The majority of them do not have adequate health information about the harmful consequences of alcohol. Alcohol and other substances of abuse were openly displayed, sold and consumed at the study sites. There were poor law provision and enforcement of laws prohibiting open display of alcohol and other substances.

**Conclusion:**

A high proportion of social drinkers in outdoor bars require intervention for their drinking behaviour. This is important because they have little or no information about the health hazards associated with excessive drinking. Presentation of these findings should contribute to increased awareness and improved response from the policy makers.

## Background

Alcohol consumption is embedded in Nigerian culture [1, 2] and most individuals drink accompanied [3], because they prefer drinking in contexts in which they find people like themselves [4].There are indications that there has been a rapid increase in alcohol consumption across all age groups in Nigeria [5]. The unrecorded alcohol consumption was estimated to be 3.5 L pure alcohol per capita for population older than 15 in Nigeria for the year after 1995 [3]. Alcohol consumption continues to increase in Nigeria as a result of the aggressive marketing activities of the leading players, and drinking is widely considered a part of social activities [3], in outdoor-open space such as at motor-parks [6, 7], by road sides and street corners [8].

Despite research evidence indicating that excessive alcohol consumption is associated with physical, psychological and social consequences [4, 9, 10], most epidemiological surveys on alcohol consumption have been household surveys, or studies carried out among population such as those in treatment facilities [11], prison population [12], adolescents [13] and the elderly [5]. These studies do not take into account the alcohol consumption, among other population such as outdoor bars, some of which groups are more likely to be involved in heavy drinking [14] and may be associated with more grave social consequences such as injuries and road accidents [6, 15]. In addition, the use of structured survey instruments is likely to influence the adequacy of survey measures and other contextual factors of alcohol consumption [16].

We already had an insight into the prevalence of alcohol consumption and alcohol use disorders among outdoor drinkers in public open places in Nigeria by a quantitative study (Under Review). However, to develop tailored interventions for this population, a qualitative assessment was needed. Prevalence, correlates and predictors of alcohol consumption and alcohol use disorders among patrons of outdoor bars in Nigeria may not be revealed with quantitative data alone, more so, the tendency to opt for such locatons is on the increase in Nigeria [8] and factors responsible for this are largely unknown. One potential reason may be lack of laws prohibiting open display, sale and consumption of alcohol in open places [6].

Thus, there is the need to examine the magnitude of alcohol consumption and its correlates in settings that are likely to be missed in epidemiological surveys such as open places using qualitative methods of assessment. This is believed to yield more robust information on the contextual factors responsible for drinking in outdoor bars.

The aim of the present paper was to use qualitative methods to assess the following: a) pattern and preferred alcohol beverages and the reasons for the preference among patrons of outdoor bars, b) perceived safe quantity of alcohol, c) presence of alcohol use legislations, and d) reasons for the continued popularity of drinking in outdoor bars.

## Methods

### Setting of study and background information on the area

This is the report of a qualitative part of a larger study that utilized a mixed quantitative and qualitative method of assessing the “Prevalence of Alcohol Consumption and Alcohol Use Disorders among Outdoor Drinkers in Public Open Places in Nigeria.” The study took place in July 2015 in Ibadan, Nigeria. The city is located in the southwestern part of the country. It has a population of about 2.5 million people as of 2009. Ibadan is divided into 11 local government areas [17].

### Sampling technique and procedure

**Fig. 1:**
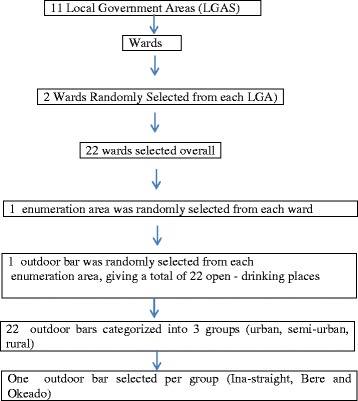
Sample Selection Flow Chart

This consists of focus group discussions (FGDs) and direct observation (DO). In this study, 22 outdoor bars were selected using as a systematic sampling method. The process of selection is as follows: all the 11 local governments in Ibadan were divided into wards, a ward being a local authority area, typically used for electoral purposes [18], then, 2 wards were randomly selected in each of the local governments (Fig. 1). The wards were then divided into enumeration areas (smallest geographical units for an enumerator to cover in order to administer a questionnaire) [18]. One enumeration area was randomly selected from each of the wards, and one outdoor bar was randomly selected from each enumeration area (names were drawn from a hat). Thus, a total of 22 outdoor bars spanning the 11 local governments in the state was selected. A list of licensed business premises with the state ministry of commerce and with the respective local government was obtained, but none was licensed to sell alcohol in open places.

All the selected outdoor bars were then categorized into three groups, urban, semi-urban and rural groups. This classification was based on local government funding allocation from the central government. One outdoor bar was then selected per group for the qualitative assessment.

In each of the three selected sites, one alcohol vendor was asked to act as gatekeeper for purposeful sampling. They selected eligible individuals, 18 and 75 years who were regular patrons of these three open-drinking places. The selected individuals were a subsample of those who took part in the quantitative assessment.

The gatekeepers were asked to explicitly consider the different educational and cultural backgrounds of the patrons in order to obtain maximum variation sampling. The demographic data of participants were reviewed throughout the recruitment process in order to ensure inclusion of different educational and cultural backgrounds. Exclusion criteria were: first-time patrons of the outdoor bar and being unable to converse in English or pidgin-English. The gatekeepers contacted existing patrons of the open-drinking places to identify those who could be interested in the study.

The investigator explained to the interested participants that the qualitative assessment was a further attempt at obtaining an in-depth understanding of open-place drinking. A sociodemographic questionnaire was administered to all prospective participants before the commencement of the qualitative study.

In total, 64 participants (49 men and 15 women) agreed to participate. The methods and procedures used in each site are summarized in Table 1. Details are available in the individual reports.

**Table 1:** Summary of FGD method

Location	Methods	Sample Size for	Composition	Sample characteristics	Sample selection	Duration
Ina-straight	FGD	6;6;8	Civil servants, professionals, retired men and women, vendors, members of the law enforcement agencies	Gender: female and male Age: 18–71 Ethnicity: Hausa, Yoruba, Igbo, Middle belters, minority tribes	Mix of purposive pre-selection by vendors and snowball sampling	90–120 min
Bere	FGD	6;6;9	Commercial drivers, some middle-class workers and traders	Gender: female and male Age: 32–61 Ethnicity: Hausa, Yoruba, Igbo, Middle belters, minority tribes	Mix of purposive pre-selection by vendors and snowball sampling	90–120 min
Oke-ado	FGD	8;8;7	Civil servant	Gender: female and male Age: 35–51 Ethnicity: Hausa, Yoruba, Igbo, Middle belters, minority tribes	Mix of purposive pre-selection by vendors and snowball sampling	90–120 min

*FGD* focus group discussion

The focus group discussion (FGD) utilized some items as contained in Alcohol Use Disorder Identification Test (AUDIT) [19, 20], as well as from extensive review of literature on the subject matter as opening questions.

As in other qualitative research in the area of addiction, the study aimed at cultural but not demographic representativeness [21]. A range of patrons of the outdoor bars from the major ethnic and language groups as well as different ages participated.

### Data collection

Three sets of FGDs (6–8 participants in each set) were conducted. Each session lasted 90 to 120 min and took place in a quiet apartment separate from the outdoor bars. The sample size was determined by using the principle of ‘pragmatic redundancy’ where data collection was stopped when it was believed that core cultural beliefs had been represented when no new information was found (data saturation) [22]. The preferred alcohol beverage and reasons for the preference, perceived safe quantity of alcohol, possible harms, alcohol use legislations and reasons for the continued popularity of open-place drinking was explored. A pre-tested focus group discussion guide was used (Table 2) and focus groups were audio-taped. The moderator was the PI who is an addiction specialist, versatile on the topic.

**Table 2:** Focus Group Discussion Guide

Questions	Focus Group Discussion Guide
	Engagement questions
1	What is the common alcohol beverages consumed in this place?
2	What do you notice or experience when you take any of these alcohol beverages?
	Exploration questions
3	What quantity of alcohol do you take at a sitting?
4	What are the harms associated with excessive drinking?
5	What are the factors are responsible for your choice of drinking place?
6	What is your opinion about the sale of alcohol in open places?
7	Are you aware of any legislation about hours of sale or open display of alcohol or sale of alcohol in open places?
8	What are the emerging trends of alcohol beverages?
9	What are your comments about the cost of alcohol?
	Exit question
10	Is there anything else you would like to say about why you take alcohol?

### Ethical consideration

Participants provided written informed consent and ethical approval was obtained from the ethical review committee, department of research, planning and statistics, Ministry of Health, Oyo State Nigeria in accordance with the Helsinki declaration.

### Focus group discussion

Each session commenced by welcoming all participants and appreciating them for participating. This was followed by the introduction of the moderator, the purpose of the FGD and the ground rules. The ground rules were: participants did the talking, there were no “yes” or “no” answers, information divulged would be kept confidential, and all activities would be audio recorded. All the other sessions, including activities, body language and other subtle clues would also be recorded.

The participants were asked to express their impression about the topic of discussion. Using open ended questions, each question on the FGD guide was discussed. The participants had the opportunity to highlight aspects of the items of discussion which seemed to be most important to them. Snacks were served at each session and free blood pressure measurement was also carried out as incentives.

The investigator encouraged participants to ask any other question that should be discussed. Participants’ who answered with a nod were encouraged to verbalize their answers for the purpose of audio recording.

Immediately after all participants had left, the principal investigator (PI) and other co-investigators had a quick debriefing while the recorder was still running. All tapes and notes were labelled with the date, time and name of the group.

The interview was conducted by the PI and his research assistants (senior registrars in psychiatry), who had been involved in qualitative research. The PI had no relationship with the respondents prior to the interviews.

All interviewers received 3-day training before the commencement of the study, to ensure strict adherence to research protocols. The method of data collection for the FGD is summarized in Table 3.

**Table 3:** Summary of Findings from Direct Observation (Study Sites *n* = 3)

Available	Site 1	Duration (minutes)	Frequency in 2 h	Site 2	Duration	Frequency in 2 h	Site 3	Duration	Frequency in 2 h	Lowest Cost (US dollars)
Beer	3+	5–7	4 times	3+	5–7	4 times	3+	5–7	4 times	0.55/50 cl
Strong beer	3+	5–7	4 times	3+	5–7	4 times	3+	5–7	4 times	0.83/50 cl
Local spirit	2+	5–7	4 times	3+	5–7	4 times	3+	5–7	4 times	0.13/5 cl
Local spirit with herbs	2+	5–7	4 times	3+	5–7	4 times	3+	5–7	4 times	0.13/5 cl
Palm wine	2+	5–7	4 times	2+	5–7	4 times	2+	5–7	4 times	0.27/50 cl
Distilled Spirit	3+	5–7	4 times	3+	5–7	4 times	3+	5–7	4 times	1.3–13/50 cl
Red wine	3+	5–7	4 times	3+	5–7	4 times	3+	5–7	4 times	1.3–13/50 cl
Tobacco	3+	5–7	4 times	3+	5–7	4 times	3+	5–7	4 times	0.55–0.75/24 sticks
Cannabis	1+	5–7	4 times	2+	5–7	4 times	3+	5–7	4 times	0.1/wrap
Prescription medication	1+	5–7	3 times	2+	5–7	3 times	3+	5–7	3 times	Variable
Food or snacks	3+	5–7	4 times	3+	5–7	4 times	3+	5–7	4 times	0.22/portion
Music	3+	5–7	4 times	3+	5–7	4 times	3+	5–7	4 times	N/A
Television	3+	5–7	4 times	3+	5–7	4 times	3+	5–7	4 times	N/A

3+: openly displayed, purchased and used by patron; 2+ available on request; 1+ only sold to known customers; Site 1: Ina-straight (Urban), Site 2: Okeado (Semi-urban), Site 3: Bere (Rural)

### Direct observation

A direct observation of alcohol sales activities (display, purchase and consumption) in the outdoor bar was also noted. Of interest were types of alcohol beverage consumed by patrons, the quantity of alcohol consumed at a sitting, gender difference in alcohol consumption, whether they were openly displayed or not, the price of the various alcohol beverages, cross-sectional view of other activities such as recreational facilities such as music, sitting arrangements, sale of snacks or food and sale of other psychoactive substances. The researchers were allocated different roles during the direct observation, such as the specific alcohol beverages to observe for, observing the availability and sale of other substances of abuse, cost of purchase of food and drinks, presence of other recreational activities. In order to avoid distraction, researchers observed for 5 to 7 min at varying frequencies. Areas that required clarification, such as the venue, display of beverages (not bar guests) were video recorded (Table 3).

### Analysis

Data analysis, commenced during the period of data collection in the field. For the FGD, the data were initially collated into broad themes in a matrix by the researcher and then transcribed at the end of each day to identify emerging themes that were explored during further focus groups with participants. A further thematic analysis was conducted aimed at unitizing the data. This was carried out by two independent analysts.

The analysis included categorizing of themes, identification of associations between themes and subthemes, search for outlier examples, intervening variables and triangulation with other data sources. The two analysts then reconciled their data and thereafter came to a consensus opinion on issues of discussion. Quotes were also reported after the data collection. Socio-demographic data were analyzed through descriptive statistics (Table 4).

**Table 4:** Sociodemographic Characteristics of Participants

	Number	Percent
Age group		
< 25	19	29.7
25–34	17	26.6
35–44	16	25.0
45–54	7	11.0
55–64	4	6.3
> 64	1	1.6
Gender		
Male	49	76.6
Female	15	23.5
Education		
No Formal	7	10.9
Elementary	22	34.4
Secondary	23	36.0
Post- secondary	12	18.8
Employment		
In Employment	51	79.7
Unemployed	13	10.3
Dwelling Area		
Urban	20	31.3
Semi-urban	23	35.9
Rural	21	32.8
Marital Status		
Not Married	28	43.8
Married	36	56.3
Religion		
Christianity	53	82.8
Islam	11	17.2
Ethnicity		
Yoruba	20	31.3
Igbo	12	18.8
Hausa	2	3.1
Middle Belt	24	37.5
Minorities	6	9.9

Data from direct observation were presented as frequency and duration of observations. Analysis was carried out using the Statistical Package for the Social Sciences (SPSS) version 20.0 software.

## Results

### Results of the focus group discussion

#### Population

Participants’ characteristics are displayed in Table 4. Mean age was 34 years, 76.6% were male. Only 10.9% of the participants had no formal education, 31.3% were from urban dwellers, 35.9% were semi-urban dwellers, while the rest, 32.8% were rural dwellers, 56.3% were married (Table 4).

### Themes

Five themes emerged from the FGD. First, the social desire to drink in open place was because; it was regarded as an important means of relaxation after work.

Second, the interaction between patrons allowed discussion about their work and other issues of relevance, in settings away from their family members.

Third, alcohol is openly displayed and sold because of a lack of legislation against it. Local brews are consumed because they are cheaper.

Fourth, drinking makes people happy, especially when there is no party to attend, more so, they are not aware of any safe limit of alcohol.

Fifth, alcohol is a medicine and is not associated with any serious health problems or accidents.

#### *Focus group discussion* Ina-straight site (urban)

In attendance were alcohol patrons, vendors, members of the law enforcement agencies. An identified theme was that the social desire to drink in open place was because; it was regarded as an important means of relaxation after work. Also, the interaction between patrons allowed discussion about their work and other issues of relevance, in settings away from their family members. One of them said “*look at all streets, you will find people drinking. We prefer these open places because our wives are not there to control our drinking.*” One young man said, “*we are free of the stress given to us by our wives.*” Another person said, “*here, we meet and discuss business, and business is best discussed when you are high or tipsy.*” Another person said, “*after taking my beer, I also take some native-herbs in spirit, this enhances my libido.*”

A vendor:- The consensus among the vendors was that the business of selling alcohol was a means of livelihood. One said, “*Niger-man no dey drink alone. If una drink alone, you go suffer alone” (*in Pidgin English*).* This means “in Nigeria, we do not drink alone; we drink in groups because if you drink in groups, you enjoy life as a group. *Another man reported, “you know it is not good to drink in the presence of children, so it is better to come outside.” In any case there are no laws prohibiting us from open drinking, and police can’t do a thing because we drink together, even in their uniform.”*

Member of a law enforcement agency:- “*We are also human beings like everyone else. In actual fact, in order for us to be able to carry out some tasks, we must be high, in fact very high.*”

#### Focus Group Discussion at Bere site (Rural)

The consensus of opinion at this site was that drinking makes people happy, especially when there is no party to attend, more so, they are not aware of any safe limit of alcohol.

A trader: “drinking *had increased over time in Nigeria because it makes us happy. For me, I drink most days because I must feel happy.*”

A working-class man: “*Nigerians like parties a lot and if there is none to attend, we routinely gather here to drink and talk.*” “*There are certainly good, well prepared vegetables that are sold. The vegetables taste better than the one prepared at home; we take it with Eko* (local solidified pap prepared with maize), *after which we start drinking*.”

Another man said “*there is no limit to the quantity of alcohol one takes because it is more or less water.*” On health related issues somebody said: “*for the young ones who are new in drinking, when they are drunk, may have road accidents, but if you are a regular drinker, you will always drive safely home. I am not aware of any health consequences.*”

A vendor said “we sell to survive*; the profit on retail sale is very low.*”

*Focus Group Discussion* Oke-Ado site (Semi-urban).

The consensus of opinion is that alcohol is a medicine.

A patron said: “*Alcohol is medicine; Paul even told Timothy to drink a little wine for stomach problem. I drink red wine, and I can take 2 bottles at a sitting. My doctor told me red wine is better than beer.”*

A local alcohol brewer: “*we brew alcohol in Nigeria. It is part of our culture. We brew at home, and there is always a high demand for it.”.*

A civil servant: “A *man who doesn’t drink can kill. Alcohol makes you happy*” “*It is good to go out and mix with people, you know… When you are married, there must be a meeting point for one and his girlfriend.”*

#### Direct observation at Ina-straight site (Urban)

The Ina - straight drinking place is located in Ibadan North local government area. It is one of the largest outdoor bars in Ibadan.

We observed alcohol and other substance selling activities at this site. The alcohol beverages were openly displayed and included, beer, imported spirits, local spirit and palm wine. Although openly displayed were different brands of cigarettes, but the vendors informed us that cannabis was available only on request to specific customers.

Also displayed were large television screens for recreation. We also observed very cordial and extremely warm atmosphere, and patrons were in groups drinking, smoking and some eating “pepper soup”. In addition, a police vehicle was found parked close to the site.

#### Direct observation at bere site (Rural)

This outdoor bar is located in Ibadan central local government. It is very busy arena in the inner core of Ibadan. On observation, alcohol was freely displayed and sold. Cannabis and prescription medications and tobacco products were accessible. Music was made available, so were small television sets. At this location, the patrons consisted of commercial drivers, some middle-class workers and traders.

#### Direct observation Oke-ado site (semi-urban)

This outdoor bar is located in Ibadan southwest, local government. The site is located in the transitional part of Ibadan characterized by a busy business environment. Similar to our observation in study sites vehicles of various types were parked by the roadside. Activities such as video games, films and international soccer were made available to patrons via large screen television sets free of charge to attract customers to their stalls.

## Discussion

Social ecological studies seek to understand the trajectories of humans and the social, economic and physical environments [23]. Thus, the survey research reported herein has investigated the social and ecological relationships between those we interviewed and their physical environment, such as that which exists between alcohol patrons and outdoor bars. Thus, our findings were discussed within these contexts. Conceivably, it is often difficult to obtain adequate samples of people who drink within specific contexts from general population surveys. Therefore, location-based sampling, in which participants are recruited from those contexts, offers an attractive alternative [24].

Five themes emerged from the FGD. First, the social desire to drink in open place was because; it was regarded as an important means of relaxation after work. Second, the interaction between patrons allowed discussion about their work and other issues of relevance, in settings away from their family members. Third, alcohol is openly displayed and sold because of a lack of legislation against it. Local brews are consumed because they are cheaper. Fourth, drinking makes people happy, especially when there is no party to attend, more so, they are not aware of any safe limit of alcohol. Fifth, alcohol is a medicine.

The major themes that emerged in the current study were that participants regarded outdoor drinking as an important means of relaxation after work, where interaction with different people is enabled in settings away from their family members and also where patrons and vendors enjoy each other’s company. The participants expressed scepticism regarding the need of legislation against drinking in outdoor bars. In their opinion, the police drink with them making it difficult for the police to enforce any law on outdoor bar drinking. The overwhelming feeling was that people will continue to drink irrespective of factors that may curb drinking, because drinking makes them happy. Some participants mentioned that when the prices of alcohol became high, some drinkers changed their choice of beverage to local brews. Participants were not aware of any serious adverse effects of alcohol. Some believed they drove better when intoxicated.

A number of elements of the social context of drinking in open places have been demonstrated in this study. This is in support of the “niche theory,” which emphasizes the niche created by some drinkers by drinking in less supervised drinking contexts such as parking lots or street corners. Our findings also support the “assortative theory”, which states that drinkers take their alcohol in contexts where they find people like themselves [25]. Understandably, assortative drinking is the characteristic pattern of drinking in Nigeria. Drinking is widely considered a part of social activities in Nigeria and therefore most consumers seldom drink alone [8]. Development of harm could have been influenced by lack of health education. For example, some alcohol consumers engaged in binge drinking of red wine, because it is believed to be medicinal, without adequate information that a bottle of fortified wine contains 13–16% alcohol or 120 g of alcohol per measure, equating 15 units per bottle. Therefore, the respondent who claimed to take 2 bottles of red wine a day must have ingested 30 units of alcohol at a sitting, which is quite harmful to the body.

### Direct observation

The direct observation yielded an amount of information. First, is the frenzy atmosphere created by the availability and music and television where interesting programs are watched in groups. Second, we observed that alcohol was openly displayed, sold and consumed. This may be attributed to a lack of legislation against open display of alcohol as this is a form of advertisement [26]. We also observed that some patrons preferred local brews as their first choice of alcohol beverage. This could be adduced to the misconception by the majority of alcohol users that it is medicinal [27]. Furthermore, it was observed that tobacco use in the form of cigarette smoking was high among these open-place drinkers. The high rate of smoking in the studied population is of a huge public significance considering the number of non-smokers who would be passively inhaling cigarette smoke.

We also noted that local brews, cannabis and prescription medications were more available in the rural outdoor bar compared with the urban counterpart. Some patrons were observed purchasing and taking some prescription medications while drinking, an action that has serious implication for drug interaction and mortality.

The results of the FGD and direct observation could be described as “facts finding” as they have yielded additional information not obtained from the quantitative assessment (In Review).

Our study underscores the need for alcohol use prevention and education program for the outdoor bars patrons. This is very relevant, especially in semi-rural/rural settings where they are at a higher risk of additional psychoactive substance use. More so, patrons of outdoor bars in the rural setting may have limited access to health care, and the risk of identification of the adverse health consequences of multiple psychoactive substance use is probably low.

More work is needed in determining factors responsible for open place drinking before it evolves into problem drinking. An understanding of the complex mechanism underpinning the reward and gratification experienced through social interaction in outdoor bar drinking is fundamental in the development of effective intervention for this population. Another factor in the understanding of the aetiology of public outdoor drinking is the presence of a psychological problem, for example mood disorder [28, 29], because some of the respondents claimed they drank “to be happy”, which may be a vague indication of mood dysregulation.

#### Policy implications

Our findings have important policy implications. Laws need to be put in place and enforced regarding advertisement, manufacture or sale of alcohol in outdoor bars, labeling of the alcoholic concentration in local brews with the approval of the appropriate authority such as the National Agency for Food and Drug Administration and Control. The implementation of effective policies that will reduce harmful and hazardous alcohol consumption requires a good understanding of the policy development process and which strategies are likely to work with good public support. For example, certain World Health Organization specific policy areas: regulating alcohol marketing; restricting alcohol sales, regulation of retail sales of alcohol; alcohol taxation and controls on alcohol packaging; strengthening drinking and driving laws; strengthening health sector response; and raising political commitment [30]. Unfortunately, none of these are being implemented in Nigeria. An important strength of the current study is that it is complementary to the findings from the quantitative assessment.

One of the limitations of this study is the qualitative method of the study. Considered in light that the composition of the FGD was culturally represented, our findings may be generalizable to other social joints from other parts of the country. Social research like this also has the advantage of exploring context dependent factors responsible for drinking among this less–well studied population. Our findings also corroborate those from the quantitative assessment.

## Conclusion

In conclusion, a high proportion of social drinkers in outdoor bars require intervention for their drinking behaviour. This is important because they have little or no information about the health hazards associated with excessive drinking. Presentation of these findings should contribute to increased awareness and improved response from the policy makers.

## Abbreviations

AUDIT: Alcohol use disorder identification test
DO: Direct observation
FGD: Focus group discussion
LGA: Local government area
PI: Principal investigator
